# Respiratory Immune Responses during Infection and Pollution Inhalation

**DOI:** 10.3390/medicina59020242

**Published:** 2023-01-27

**Authors:** Cormac McCarthy, Patrick Geraghty

**Affiliations:** 1 University College Dublin School of Medicine, Education and Research Centre, St. Vincent’s University Hospital, D04 T6F4 Dublin, Ireland; 2 Department of Medicine, State University of New York Downstate Health Sciences University, Brooklyn, NY 11203, USA

The COVID-19 pandemic highlighted the importance of lung immune responses to pathogens and environmental factors. The lungs and upper airways are the first sites of exposure to external factors. In response to harmful organisms and toxic pollutants, the lungs have a comprehensive and complex system of resident and immune cells [1]. Within the lung, there is a complex interplay between resident cells, infiltrating immune cells, a multifaceted microbiome, a mucus-clearing system, and secreted immune proteins that shape the outcome of host–pathogen, host–allergen, and host–particle interactions within the lungs [2]. This research topic aimed to collect recent updates and novel findings to elucidate important changes that occur within the lungs during an infection or following exposure to noxious factors. Therefore, this Special Issue topic invited contributions to not only showcase the characteristics of immune responses and noxious and infectious inhaled agents that are linked to respiratory diseases, but also depict the underlying and potentially targetable molecular mechanisms. This issue accumulated eight articles, including three original research articles, four reviews, and one case report from a total of 42 authors from two different continents and five countries.

Five of the featured studies in this Special Issue focus on pulmonary infections or the normal lung flora. The first two studies investigate factors of the COVID-19 pandemic. A retrospective single-center observational study by Feng and colleagues [3] reviewed the health records of 271 COVID-19 patients during the first wave of the pandemic within an institution in New York City. At this early time in the pandemic and the patient population, two-thirds of these admitted COVID-19 patients died, and the investigators noted that age, blood urea nitrogen, and blood neutrophil percentage were significantly associated with mortality. They also observed that elevated procalcitonin and C-reactive protein levels were significantly associated with mortality, even when adjusting for age, sex, and race/ethnicity. Procalcitonin, a pro-hormone that plays a role in calcium homeostasis, was also found to be associated with the likelihood of intensive care unit acceptance. Procalcitonin levels are known to increase in sepsis [4] and reduce following antibiotic treatment [5]. The second COVID-19-focused study by Rodriguez-Blanco and colleagues [6] utilized a pilot randomized, controlled, parallel, double-blind, two-arm clinical trial to assess the feasibility and effectiveness of a novel therapeutic exercise program through telerehabilitation. Thirty-six patients with mild-to-moderate COVID-19 were randomized to either a rehabilitation program based on muscle conditioning or a parallel control group who did not perform physical activity. The authors demonstrated that there were significant improvements in functional tests and dyspnea scores in the experimental group and there was excellent adherence to the program. Despite the small numbers and pilot design, this study demonstrated that telerehabilitation was feasible, safe, and well-adopted in this population, indicating the potential future use of telemedicine for rehabilitation post-lung injury or infection. 

A review article by Guo-Parke and colleagues took a different perspective by focusing on the airway epithelium as a major site of anti-viral infection responses and detailed the current insights within the literature to detail the mechanisms of viral evasion and host interferon antiviral defense signaling impairment of the airway epithelium within chronic obstructive pulmonary disease (COPD) patients [7]. Viral infections were found to significantly impact COPD patient hospitalization and the continued loss of lung function [8]. Here, the authors also briefly detailed the potential role of the epithelium anti-viral responses against SARS-CoV-2 and the implications of impaired epithelial antiviral responses on therapeutic options [7]. The impact of antimicrobial therapy and the influence of environmental exposure on the upper airway microbial flora was the topic for discussion in the review article by Elgamal and colleagues [9]. The imbalance of normal microbial flora, dysbiosis, is a possible contributing factor to the initiation or progression of pulmonary diseases. The development and maintenance of a healthy upper airway microbiota are associated with normal respiratory development and function, but aging and environmental factors can trigger a shift in the microbial composition of the lungs and upper airways [10]. Elgamal and colleagues outline the role of the upper airway microbiota in preserving homeostasis and detail the impact of environmental airborne pollutants (such as cigarette smoke or particulate matter), inflammation, and preexisting diseases on the airways and their symbionts [9].

O’Reilly and Dunican reported a case series on the utility of monoclonal antibody therapy in allergic bronchopulmonary aspergillosis (ABPA) [11], a respiratory disorder occurring in response to *Aspergillus fumigatus* that can complicate the course of asthma or cystic fibrosis and is often very difficult to treat. In this case series, three patients, refractory to corticosteroids and antifungals, were treated with anti-IgE therapy (omalizumab) and subsequently with the anti-IL5R antibody; benralizumab demonstrated a clinical response in all patients. This series adds to the growing data supporting the use of monoclonal biologics in ABPA, but there remains a lack of randomized controlled trials to support this; however, ABPA is rare and such trials are logistically challenging.

The remaining three papers within this Special Issue focus on several important mechanistic players involved in pulmonary diseases and noxious inhaled factors, such as alpha-1 antitrypsin (AAT) deficiency (AATD), vaping products, and cellular senescence.

Alpha-1 antitrypsin is a serine protease inhibitor that demonstrates many immunomodulatory functions and individuals with AATD are at increased risk of early-onset emphysema and COPD. Hawkins and colleagues reported a translational study in which they assessed the expression of the membrane voltage-gated proton channel-1 (HVCN1) in AATD patients receiving exogenous AAT augmentation or not and compared it to healthy individuals [12]. They showed that HVCN1, which is integrally linked to neutrophil function, is under-expressed in neutrophils of ATTD individuals and that HVCN1 undergoes proteolytic degradation in activated neutrophils primarily due to neutrophil elastase activity which is increased in AATD. Interestingly, they demonstrated that augmentation therapy with exogenous AAT increased HVCN1 expression on neutrophils. Despite the small number of patients who received augmentation therapy, this study poses questions regarding the links between neutrophil-derived proteases and HVCN1, as well as their impact on airway inflammation in AATD and COPD.

Rivas and colleagues [13] review the COPD literature linking the potential role of cellular senescence to the pathogenesis of COPD. COPD is recognized as a disease of accelerated lung aging and multiple recent studies characterize the significance of the accumulated senescent cells in the lungs of COPD patients. In COPD, senescence is linked to telomere dysfunction, DNA damage, and oxidative stress [14]. This review manuscript details the major players in COPD and senescence, discusses their possible role in the pathogenesis of COPD, and details possible new therapeutics to target these senescent cells for the treatment of COPD [13].

Finally, O’Callaghan and colleagues review the literature regarding vaping-associated lung injury [15]. Before the recent COVID-19 pandemic, the emergence of e-cigarette and vaping-associated lung injury (EVALI) was possibly the most recent lung disease being described which resulted in an epidemic, with over 2500 cases in the USA alone in 5 months in 2019 [16]. O’Callaghan and colleagues summarized the background of e-cigarettes and vaping devices, the reasons for their increased use, as well as the mechanisms of lung injury caused by their use. In brief, they reviewed the studies which demonstrated the effects of vaping at a cellular level, including how vaping increases oxidative stress, results in endothelial cell dysfunction, and impairs host defense with functional changes in innate immune cells. This review summarized the clinical findings reported and explained that the long-term effects remain to be seen and that further mechanistic studies are needed.

In summary, this Special Issue showcases diversity in clinical and basic research focusing on lung disease and the impact of environmental and microbial exposures on immune responses within the lungs, which we hope will help stimulate further research to address a timely and relevant worldwide problem. Currently, pulmonary diseases represent a major global issue, especially with a growing aging population and the emergence of new pathogens that could trigger another pandemic.
